# Clinical value of Beery visual-motor integration and Beery VMI supplemental tests in Chinese preschoolers: a modified replication cross-sectional study

**DOI:** 10.3389/fped.2025.1531192

**Published:** 2025-03-06

**Authors:** Xin Tang, Xuan Xu, Cong Wang, Lijuan Ao

**Affiliations:** ^1^ Physiotherapy Department, School of Rehabilitation, Kunming Medical University, Kunming, Yunnan, China; ^2^ Occupational Therapy Department, School of Rehabilitation, Kunming Medical University, Kunming, Yunnan, China

**Keywords:** visual-motor integration, visual perception, motor coordination, culture variation, Chinese preschoolers

## Abstract

**Objective:**

Previous studies have indicated that visual-motor performance is affected by cultural variations. This study aimed to examine differences between the proportion of children with poor visual-motor integration (VMI) performance in published population norms with that in the sample population of this study and to assess the predictive value of the visual perception(VP) and motor coordination(MC) scores in explaining VMI variance in Chinese preschoolers.

**Methods:**

A random sample of 421 children ageds 3.0–6.11 years (mean age, 4.51 ± 0.93) participated in this study. The Beery VMI, VP, and MC were administered in sequence by qualified raters.

**Results:**

The standard scores of VMI in all age groups of this study were significantly better than those in U.S. norms. Overall, 61 (14.5% of the total sample) and 6 (1.4% of the total sample) children did poorly in the VMI test using study population norms and published population norms, respectively. The proportion of children with poor VMI performance using published norms was significantly lower compared with that using study sample population norms (*p* < 0.001). Multiple regression analysis showed that VP and MC were significantly related to the VMI (VP: *β* = 0.185, *p* < 0.001; MC: *β* = 0.400, *p* < 0.001) and 25.0% (*F* = 69.571, *dF* = 2, *p* < 0.001) variance of VMI could be explained by VP and MC.

**Conclusion:**

Our findings support that the effects of culture should be considered when interpreting the results of Beery VMI using published norms, especially in poor performance diagnosis. Our findings further support that three tests should be assessed individually during the visual perception examination regardless of cultural context. Chinese preschooler–based norms need to be established in future investigations to determine the diagnostic value of the Beery VMI.

## 1 Introduction

Visual-motor integration (VMI) refers to the degree to which visual perception and fine motor skills are well coordinated and integrated (1). It assesses a child's ability to respond with fine motor skills after interpreting visual information (2). VMI plays a critical role in the overall level of functional development in children. Participation in basic and advanced childhood daily activities such as throwing and catching balls, keyboarding, and handwriting requires VMI skills (1, 3, 4). The VMI performance of preschoolers has been examined to relate to academic performance in school-age children, such as in mathematics and reading (5, 6). Furthermore, executive function, part of cognitive function, has been shown to be significantly related to VMI both in preschoolers and school-aged children (2, 7).

VMI performance is generally lower in children with developmental disorders compared with typically developing children. School-age children with ADHD exhibit significantly lower VMI performance and are uniquely associated with math skills (8). The rate of VMI deficit is higher in preterm children, as they have been shown to have lower VMI scores compared with term-born children (9). Similar results can also be detected in children with ASD (10), Williams syndrome (11), cerebral palsy (CP) (12), among others. Then, it is likely a common deficit in developmental disorders. However, some researchers have reported that therapy could improve VMI performance (13). Therefore, it is essential for clinical practice to provide the accurate diagnostic criteria for VMI deficit at an early age. The Beery VMI is a commonly used worldwide tool for examing both the VMI deficit and the effectiveness of therapy (14, 15). It was first published in 1967 but was standardized in 2010 and published in the sixth edition of the Beery VMI Administration, Scoring, and Teaching Manual (16). The test provids the norms for participants aged 2–100 years in the United States to describe the level of VMI performance. The psychometric properties of Beery VMI have been well-documented in early studies (16).

Numerous factors have been explored in relation to the performance of Beery VMI. In terms of demographic data, chronological age was the best core variable to predict VMI development in different culture-cross research (1, 17). Meanwhile, the inconsistent results of gender differences have been suggested in previous studies (1). Additionally, even though previous study have suggested the absence of significant differences among the different ethnic groups (18), Beery stated in the manual, “At an early age, Chinese children performed somewhat better than American children on the Beery VMI” (16). This conclusion was also supported in later studies, for example, research results involving 288 healthy children aged between 3 years 6 months and 5 years 11 months in Hong Kong, revealed that the preschoolers in Hong Kong performed significantly higher than their U.S. counterparts (19). Additionally, significantly higher performances were observed among Chinese children living in Shanghai and Ningbo, both in preschoolers and school-aged children (20). The cultural influence on the VMI performance of Chinese children has also been investigated in Nanjing. The results showed that the VMI skills of children aged 5–6 years exceeded those of their counterparts in the U.S. normative samples for the same ages (21). Furthermore, among a Singaporean population, the scores of the Chinese children were significantly higher than those of Malay and Indian ethnic groups (22). These findings suggest that cultural differences exist between Chinese children and U.S. normative data. Studies have shown that Chinese children tend to achieve better scores than those in U.S. published norms. Therefore, assessing Chinese children using available published U.S. norms may lead to an underestimation of the rate of VMI deficit; hence, children with potential deficits may not receive the required therapy. However, there is limited information on the proportion of poor VMI performance between U.S. normative samples and study population samples.

In addition to demographic factors, other functional skills were related to VMI performance, including fine motor and visual perception, etc (16). Therefore, the supplemental tests, which assess visual perception (VP) and motor coordination (MC), should be performed after the VMI test. The aim of these supplements is to examine the potential causes of a child's poor VMI skills. However, in a previous study, multiple linear regression revealed that 36.2% of the variance in VMI could be explained by supplemental tests (3). The researchers defined poor performance as a score >1 standard deviation (SD) below the mean of the study population norms. A total of 35 children performed poorly on the VMI test, and only 17.1% (6 children) performed poorly both in the VP and MC tests. However, 20% (7 children) and 14.3% (5 children) performed poorly in VP and MC alone separately, while 48.6% (17 children) performed normally in both the VP and MC tests. The percentage of variance explained by the supplemental tests was even lower, only 22% in the later modified replication study (23). However, there is limited research to examine the value of supplemental tests in assessing VMI's poor performance. Moreover, the clinical value of these supplemental tests in populations of Chinese children has not yet been investigated.

Therefore, the first purpose of this study was to examine the differences in VMI performance between Chinese preschoolers and their counterparts in U.S. normative data and the differences in the proportion of children with poor VMI performance between study sample population norms and published population norms. We also modified the protocols of previous studies in terms of population ethnicity and age to explore the correlations between VMI, VP, and MC scores and the predictive value of VP and MC scores in explaining VMI variance in Chinese preschoolers.

## 2 Methods

### 2.1 Design

This cross-sectional study was conducted to examine the baseline data from a randomized controlled trial. This study was approved by the Medical Ethics Committee of Kunming Medical University (KMMU2023MEC147) and was registered in the Chinese Clinical Trial Registry. (registration number: ChiCTR2300074122). The parents or legal guardians of the participants provided written informed consent at the time of enrollment.

### 2.2 Participants

A total of eight kindergartens in Kunming, Yunnan province, were selected using a random sample method. A random sample comprising 421 children [215(51.1%)male and 206(48.9%) female children] ages 3.0–6.11 years (mean age 4.51 ± 0.93 years), from the selected kindergartens participated in this study. Children were excluded from the study if (1) they were defined as “abnormal” in the Denver developmental test II (24); (2) their teachers identified physical or intellectual or developmental disabilities which led to them being unable to complete the assessment.

### 2.3 Procedures

The Beery VMI and supplemental tests were administered according to the Beery VMI Administration, Scoring and Teaching Manual (6th Edition) in the classroom by one physical therapist and one occupational therapist of the research team from August 27, 2023 to April 3, 2024. Both testers were trained in the methodology of the three tests. The children ageds 3.0–3.11 years were administered the tests individually. Children ages 4.0–6.11 years were arranged in groups of five for each tester. All tests were scored by an experienced researcher who was also in charge of transforming raw scores to standard scores and percentile ranks.

### 2.4 Measures

The Beery VMI (6th edition) Ages 2 through 7 (SHORT FORM), the Beery VMI Developmental Test of Visual Perception (VP test), and the Beery VMI Developmental Test of Motor Coordination (MC test) were sequentially administered to the participants. The Beery VMI is a norm-referenced measure that could be used to examine VMI performance in individual aged 2–100 years (16). The short form version is for children aged 2–7 years and includes a sequence of 21 geometric shapes for VMI testing, and 30 geometric shapes for supplemental tests. In the Beery VMI test, the participants were required to copy 21 geometric shapes in a booklet using a pencil from simple to more complex shapes. The participants were then shown the target geometric shapes and were instructed to select the correct one from 2 to 7 options in the VP test. Finally, the participants were asked to connect the dots within paths to form specific geometric shapes in the MC test within 5 min. All three tests were completed in the above sequence.

### 2.5 Data analysis

SPSS version 25.0 (IBM Corp., Armonk, NY, USA) was used for statistical analysis. We tested the normality and homogeneity of variances to ensure that no assumptions were violated. A one-sample t-test was used to compare the mean standard score differences in VMI performance in all age groups between Chinese participants and those in U.S. published norms (mean standard score ± SD, 100 ± 15) in the first step of the first objective. In terms of differences in the proportion of poor performance between the study sample population norms and published population norms, according to the previous studies, we defined poor performance as a score below the 16th percentile when using the published norms and a score >1 SD below the mean when using study sample population norms. Afterwards, the Pearson Chi-square was conducted to examine the difference. However, Fisher's Exact Test was performed if any cell had an expected count of less than 5. Both the Pearson correlation coefficient and multiple linear regression were conducted for the second objective. We used the Pearson correlation coefficient to explore the relationship between the standard and raw scores of three tests. Multiple linear regression was performed to examine the predictive value of VP and MC in explaining VMI variance. A VIF value blow five and a collinearity tolerance value of 0.65 and above indicated that the variables were not multicollinear. The level of significance for this study was established at *p* < 0.05.

## 3 Results

Table 1 shows the mean raw scores, mean standard scores, and SD of all the age groups. In three tests, the participants of all age groups performed higher than those in published norms (mean ± SD, 100 ± 15). The raw scores of three tests increased along with age, which coincided with developmental milestones (age 3.0–3.11 years,10.57; age 4.0–4.11,13.31 years; age 5.0–5.11 years,16.06; and age 6.0–6.11 years,17.52). The mean raw scores and standard scores of the VP test were consistently high across all age groups. However, the standard deviation (SD) of the VP test was relatively larger, which was consistent with the findings of previous studies. Descriptive statistics revealed that the sample means and SDs of standard scores for all three tests were indicative of normal distribution and age appropriateness.

**Table 1:** Descriptive statistics for visual motor integration (VMI) visual perception (VP) and motor coordination (MC) of total Chinese preschoolers.

Tests of age groups	*n*	Mean raw score	Mean standard score	Standard deviation of standard score	95% confidence interval for standard score
VMI	421	14.51	106.36	8.184	105.58–107.15
VP	421	17.61	110.42	11.796	109.29–111.55
MC	421	15.82	105.35	10.216	104.35–106.31
3.0–3.11 years	77				
VMI		10.57	106.95	6.669	105.43–108.46
VP		13.05	109.56	13.492	106.50–112.62
MC		11.52	103.53	11.710	100.87–106.19
4.0–4.11 years	133				
VMI		13.31	107.64	8.496	106.18–109.10
VP		16.49	109.08	12.125	107.00–111.16
MC		14.79	104.57	9.525	102.94–106.21
5.0–5.11 years	153				
VMI		16.06	106.14	8.709	104.93–107.70
VP		19.18	111.35	11.259	109.57–113.13
MC		17.37	107.22	10.709	105.50–108.90
6.0–6.11 years	58				
VMI		17.52	103.24	7.104	101.74–105.50
VP		21.10	114.00	10.273	111.37–116.63
MC		19.05	106.79	9.492	104.36–109.22

As showen in Table 2, the standard scores of the VMI in Chinese preschoolers were significantly higher than those in U.S. norms in all age groups. The most significant difference was in the 4.0–4.11 age group (mean difference 7.639), while the smallest difference was in the 6.0–6.11 age group.

**Table 2:** Means and SD of Beery VMI standard scores between Chinese and U.S. preschoolers.

Age groups	Chinese preschoolers	U.S. preschoolers	Mean difference	95% confidence interval of the difference		*t*	*p*
	Mean of standard scores ± SD	Mean of standard scores ± SD		Lower	Upper		
3.0–3.11 years	106.95 ± 6.669	100 ± 15	6.948	5.43	8.46	9.143	<0.001
4.0–4.11 years	107.64 ± 8.496	100 ± 15	7.639	6.18	9.10	10.369	<0.001
5.0–5.11 years	106.14 ± 8.709	100 ± 15	6.137	4.75	7.53	8.717	<0.001
6.0–6.11 years	103.24 ± 7.104	100 ± 15	3.241	1.37	5.11	3.475	0.001

The differences between the proportion of poor performance of VMI in published population norms and the sample population norms are shown in Table 3. According to the definition of poor performance in Beery-VMI, we defined a percentage rank below the 16th-percentile as poor performance in published population norms and score >1 SD below the mean VMI in sample population norms of this study, respectively. The results showed that six children had poor VMI scores in published population norms, whereas 61 children performed poorly in the VMI norms of the sample population. Fisher’ s exact test analysis showed that the proportion of children with poor VMI scores in published population norms was significantly lower than that in the sample population norms (*p* < 0.001).

**Table 3:** Differences in poor performances in VMI between published population norms and sample population norms.

Published population	Sample population		total	*p*
	Poor performance	Normal performance		
Poor performance	6	0	6	
Normal performance	55	360	415	
Total	61	360	421	<0.001

Regarding the second objective, significant Pearson correlation coefficients among the standard scores of three tests were examined and presented in Table 4 (*r* = 0.330, *p* < 0.001; *r* = 0.467, *p* < 0.001; and *r* = 0.366, *p* < 0.001, for VMI&VP, VMI&MC, and VP&MC, respectively). The results were similar to those reported by previous studies. However, the correlation coefficients among the raw scores of three tests was found to be stronger than those reported by previous studies (*r* = 0.731, *p* < 0.001; *r* = 0.784, *p* < 0.001; and *r* = 0.686, *p* < 0.001for VMI&VP, VMI&MC, and VP&MC, respectively).

**Table 4:** Correlations among VMI, VP, and MC scores.

Score category	VMI and VP	VMI and MC	VP and MC
Sample standard score	0.330**	0.467**	0.366**
Sample raw score	0.731**	0.784**	0.686**

** *p* < 0.001.

Furthermore, the results of the multiple regression analysis showed that the standard scores of supplemental tests were significantly related to the VMI (VP: *β* = 0.185, *p* < 0.001; MC: *β* = 0.400, *p* < 0.001) as shown in Table 5. Additionally, 25.0% (*F* = 69.571, *dF* = 2, *p* < 0.001) of the variance in VMI could be explained by VP and MC. Regarding the analyses using the sample population norms, poor performance was defined as a score >1 SD below the mean, as suggested in previous studies. The results indicated that a total of 61 children (14.5% of the total sample) performed poorly on the VMI test. Out of these, only seven children (11.5%) demonstrated poor performance on both the VP and MC tests. Additionally, nine children (14.8%) performed poorly on the VP test alone, while 17 children (27.9%) performed poorly on the MC test alone. Surprisingly, 28 children (45.9%) performed within the normal range on both supplemental tests.

**Table 5:** Result of multiple linear regression in predicting VMI (SS) variance by VP and MC.

Variables	*B*	*SE*	*β*	*t*	*p*	*VIF*
Constant	58.366	4.144		14.083	<0.001	
VP (SS)	0.129	0.032	0.185	4.056	<0.001	1.163
MC (SS)	0.321	0.037	0.400	8.749	<0.001	1.163
*R* ^2^	0.250					
*F*	69.571					
*p*	<0.001					

SS, standard scores.

Furthermore, when applying the published norms, it was observed that six children (1.4% of the total sample) performed poorly on the VMI test. Among these, two children (33.3%) also demonstrated poor performance on the MC test, while none of them performed poorly on the VP test. Interestingly, four children (66.7%) who performed poorly on the VMI test had scores that were within the normal range on both supplemental tests.

## 4 Discussion

This study aimed to investigate the cultural effect of the diagnostic value of Beery-VMI on poor performance and the clinical value of supplemental tests in explaining VMI variance in Chinese preschoolers. Overall, Chinese preschoolers in all age groups achieved higher scores in Beery-VMI when compared to those of U.S. norms. Furthermore, our hypothesis was supported by a significant difference in the proportion of poor VMI performance between using study sample population norms and U.S norms. Moreover, only a 25% of the variance in VMI performance could be explained by supplemental tests, which was similar to what was reported in a previous study (23).

Though some culture-cross studies indicated that the Beery-VMI is a culture-free assessment tool (17, 18), other previous studies involving Chinese children from Hong Kong (25), Shanghai, and Ningbo cities (20) consistently reported significantly higher scores compared to U.S. norms, particularly among preschoolers. Although our participants lived in Kunming, located in Southwest China, with relatively lower socioeconomic development, the results of our study were similar to those of previous conclusions. This is probably due to the similar cultural context. Numerous factors related to cultural variance have also been discussed in previous studies (22). For example, learning and writing Chinese characters improved VMI performance because the characters composed distinct brush strokes and specific structures similar to shapes in assessment booklets (25). Moreover, children in China begin their kindergarten lives at 3 years of age or even younger, and activities in kindergartens, such as painting, and writing, practice basic VMI skills. Daily living factors such as using chopsticks, have also been considered as potential causes (26). However, our findings provide further evidence that cultural variance should be considered when performing the Beery VMI test, as it is a form of a norm-referenced test tool.

Although several studies have examined the differences in VMI performance between two ethnic groups (3, 22), few have investigated the difference between poor performance defined in study population sample norms and published population norms. One role of a norm-referenced standardized assessment tool is to document a child's developmental and functional status compared with those of peers in the same culture context (27). This ensures that children at risk are identified and given the required support as early as possible. In our study, 61 children with scores >1 SD below the mean of the study population norm were found to have poor performance in VMI. In this study, 14.5%(421 children in total) of the participants had poor performance of VMI scores, which is similar to the 18.1% (35 children with poor performance in total 193) and 15.5% (11 children with poor performance in total 71) reported in studies conducted separately by Kulp et al. and Avi-Itzhak et al. (23, 28). This result implied that the Beery VMI was sufficient to examine the poor performance of Chinese preschoolers; therefore, it could be used generally in clinical and educational institutions for early developmental stage screening. However, only six children in this study were found to perform poorly using published population norms. There are significant differences between the two proportions. This suggests that Chinese children who had normal scores in Beery VMI using published population norms may have performed differently from their peers. In other words, the rate of VMI poor performance may be an underestimation in Chinese children if using published population norms. Meanwhile, the VMI ability has been examined to predict handwriting accuracy, speed, and academic achievements in primary school (3, 4, 6, 29). Preschoolers with poor VMI scores may have more challenges in future development. Additionally, the diagnostic value of the norm-referenced test should be interpreted in the same cultural context if the functional skills assessed using this test are influenced by cultural differences. Therefore, the norm values of the Beery VMI involving Chinese children, especially preschoolers should be established to estimate the functional status of children in future research.

Regarding our second objective, we modified research conducted by Kulp et al. and Avi-Itzhak et al. to examine the clinical value of supplemental tests in explaining VMI variance in Chinese preschoolers (23, 28). In this study, significant correlations among the Beery VMI and supplemental tests were identified using Pearson correlation coefficients from the participants’ raw scores indicating that the abilities assessed by the three tests are interrelated. These findings also support the inclusion of the supplemental tests as a part of the Beery VMI assessments and align with the results of replication studies. Additionally, a significant correlation was also be detected in standard scores among the three tests in this study. The standard scores could be interpreted into seven different qualitative categories. For example, the child would be categorized into the “Above Average” group if the standard score was between 110 and 119, whereas the “Below Average” group was composed of the children with standard scores between 80 and 89. These categorizations would help caregivers better understand each child's performance and provide valuable information for determining whether a child should receive interventions. Therefore, standard scores are commonly used in clinical practice. The significant correlation among these standard scores indicated that the category indicators of the three tests were interrelated. In the construct validity research of Beery VMI, the coefficient between VMI and MC(*r* = 0.56) was higher than that between VMI and VP (*r* = 0.48) in children aged 1–7 years (16); a similar trend was also observed in this study (*r* = 0.467, *p* < 0.001; *r* = 0.330, *p* < 0.001 for VMI & MC, VMI & VP separately). The children enrolled in this study were required to complete both VMI and MC tests that required writing, and the same shapes were used in both tests, this may be the potential cause of a stronger coefficient. The motor control requirements in both Beery VMI and MC tests emphasize the importance of handwriting ability. While handwriting plays a crucial role in the development of fine motor skills (30), it is important to note that there are various activities and tasks, such as block building and bead threading (31), that can be employed to assess hand coordination and in-hand manipulation beyond solely relying on handwriting tasks. For example, there are other hand manipulation items besides writing tasks in the visual motor integration subtest of Peabody Developmental Motor Scale-2 (PDMS-2) (30). Interestingly, whether VMI is more related to handwriting skills or general hand manipulation skills is yet to be investigated. Therefore, different outcome measures, such as PDMS-2, could be used to further clarify the correlation between VMI and general hand manipulation skills besides handwriting skills in future studies. However, the finding of our study suggest that preschoolers with delayed motor ability rather than visual perception ability may be at higher risk of poor performance in VMI. Meanwhile, the results also indicated that the VMI performance of children with motor impairments caused by neurological disorders, such as cerebral palsy and developmental coordination disorders, likely deserves more attention.

VMI performance is related to several factors, both in the personal factor domain and the body structure and function domains based on the International Classification of Functioning, Disability and Health model. For example, age was the core demographic factor in the personal factor domain because VMI skills are developmental (1). Other factors, including gender and race, also influence VMI performance. However, most personal factors are non-modifiable; therefore, modifiable factors in the body structure and function domain deserve more attention from occupational therapists in clinical setting. The statement of the Beery VMI administration indicated that the supplemental tests of Beery VMI provide potential modifiable factors to explain the VMI deficit (16). The clinical value of supplemental tests has been investigated in population of American children. Kulp et al. reported that a 36.2% variance in VMI performance could be explained by supplemental tests in school-age children (28), whereas it only explained 22% of the variance in children between 4 and 5 years of age in Avi-Itzhak et al. replication research (23). Our study extended to children between the ages of 3 and 6.11 years, and our result showed that 25% of the variance in VMI performance could be explained by the two supplemental tests. On the other hand, it suggested that a large proportion of variance was likely explained by integration between visual perception and motor ability rather than VP or MC alone. This observation could be supported partly by our results. Furthermore, 61 children were identified as having poor VMI scores in the sample population norms, whereas 28 (45.9%) of these participants had normal scores both in the VP and MC tests. This demonstrated that a child identified as normal both in VP and MC but still likely at risk of poor VMI performance. All these results support the part-whole hierarchy hypothesis in Beery VMI construct validity research (16). It has also been hypothesized that the VMI test is more demanding than the VP or MC test alone. These findings provide further evidence that three tests should be tested individually to clarify the specific deficit area and to promote effective intervention programs.

Additionally, the results of our multiple regression analysis revealed that both MC and VP performance were significant predictors of VMI in this study model. Meanwhile, MC performance was the stronger predictor rather than VP in our predicting model (MC: *β* = 0.400, *p* < 0.001; VP: *β* = 0.185, *p* < 0.001). These results are consistent with those reported by as study on school-age participants (28). However, these findings are in contrast with the result reported by Avi-Itzhak et al., in which, MC was not a significant predictor in their predicting model (23). They found 11 children with poor VMI performance but none of them performed poorly on the MC. However, 17 children (27.9%) with poor VMI performance did only poorly on MC alone in the current study. The difference in the proportion of poor MC performance in children with poor VMI performance may result in a different conclusion. It has been suggested that preschoolers who performed better in the MC test likely achieved better performance in the VMI test. The significant correlation between fine motor skills and cognitive functions has been examined in recent extensive research (31, 32). Fine motor skills, especially at an early age have been shown to predict not only cognitive functions, such as mathematics (33), but also visuospatial deductive reasoning in adolescence (34). Our finding extended the evidence, whereas the developmental connection between fine motor coordination and VMI ability remains to be investigated. Although limited findings have indicated that children with low motor ability have lower VMI scores, their perceptual skills remain unaffected (35). The results of these tests could help therapists to design specific intervention programs, especially for children with motor impairments. For example, the VMI performance of children with neurological disorders such as cerebral palsy improved after receiving physical therapy exercises that facilitated hand-eye coordination and fine motor skills (31). In our previous analysis, the coefficient between VMI and MC was more substantial than that between VMI and VP. Therefore, both the coefficient of correlation among three tests and the predictors in multiple regression analysis indicated the critical correlation between MC ability and VMI performance in preschoolers.

This study has several limitations. First, the lack of diagnostic studies on VMI performance in Chinese preschoolers restrains our capacity for comparison. Second, previous studies have reported demographic factors that related to VMI performance. Incorporating these factors may improve the explained variance proportion in predictive model. Third, our sample was composed mainly by children from city in southwest China. Therefore, the generalizability of the findings may be limited. Despite these limitations of this study, our findings provide valuable information on the diagnostic value of Beery VMI for Chinese preschoolers. As a result, this study extends the research finding on cultural effects on Beery VMI tests.

## 5 Conclusion

Our study found that children living in the context of Chinese culture exhibited functional deficits that may not have been identified using the published population norms of the Beery VMI assessment. Cultural factors should be taken into consideration in the evaluation of the VMI performance. Moreover, the lower variance of VMI could be explained by VP and MC; therefore, these three tests should be assessed individually during clinical examination, irrespective of cultural context. To ascertain the diagnostic significance of the Beery VMI assessment, it is imperative to establish norms specific to Chinese preschoolers in future investigations.

## Data Availability

The original contributions presented in the study are included in the article/Supplementary Material, further inquiries can be directed to the corresponding author.
